# Whipple’s disease with multiple serous effusions as the clinical manifestation: a case report and literature review

**DOI:** 10.1186/s40249-026-01441-w

**Published:** 2026-04-13

**Authors:** Luan Zhang, Tianyi Wu, Nannan Xu, Gang Wang, Lintao Sai, Lili Wang, Hui Yang, Shanshan Wang, Ke Xu

**Affiliations:** 1 Department of Pediatric, The Second Hospital, Cheeloo College of Medicine, Shandong University, Jinan, 250033 Shandong China; 2 Department of Infectious Disease, Cheeloo College of Medicine, Qilu Hospital, Shandong University, Jinan, 250012 Shandong China

**Keywords:** Whipple’s disease, *Tropheryma whipplei*, Multiple serous effusions, Immune reconstitution inflammatory syndrome

## Abstract

**Background:**

Whipple’s disease, caused by *Tropheryma whipplei*, is a rare multisystem infectious disorder with diverse clinical manifestations. Typical symptoms include arthralgia, nausea, vomiting, diarrhoea, and weight loss, while nonspecific features such as fever, anaemia, and lymphadenopathy may also occur. Presentations with multiple serous effusions are exceedingly rare. Diagnosis remains particularly challenging in resource-limited regions because of nonspecific symptoms and limited access to advanced diagnostic techniques.

**Case presentation:**

A 34-year-old male presented with fever, vomiting, diarrhoea, mild dry cough, and anorexia. Whole-body computed tomography revealed systemic inflammatory changes that mimicked vasculitis, including multiple serous effusions (pleural, peritoneal, pericardial, and pelvic) and omental thickening. Initial anti-infective therapy failed. Serological tests, pleural fluid analysis, and thoracoscopic pleural biopsy excluded systemic vasculitis and pleural malignancy. Given the diagnostic uncertainty, subsequently 18F-fluorodeoxyglucose positron emission tomography demonstrated diffuse peritoneal thickening accompanied by hypermetabolism, which prompted a biopsy. Definitive diagnosis was achieved via laparoscopic omental biopsy with histopathology, periodic acid–Schiff staining, and polymerase chain reaction. Despite receiving targeted antibiotic therapy for *T. whipplei*, the patient showed suboptimal clinical improvement. We speculated that the recurrence of the patient’s condition was more likely attributable to immune reconstitution inflammatory syndrome. Combination therapy with doxycycline, hydroxychloroquine, and short-term glucocorticoids induced sustained remission. After more than 2 years of targeted anti-*T. whipplei* therapy, the patient demonstrated a favourable recovery.

**Conclusions:**

Multiple serous effusions are uncommon clinical manifestations of Whipple’s disease. Early identification of *T. whipplei* infection and timely targeted therapy are critical for improving patient prognosis.

**Graphical Abstract:**

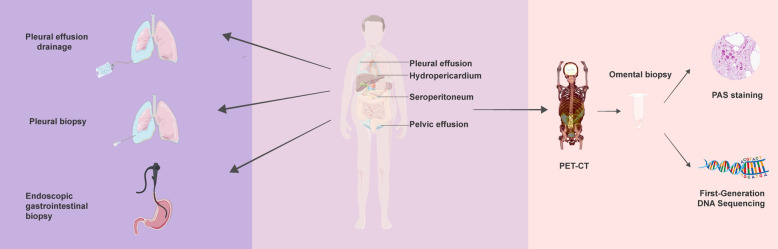

**Supplementary Information:**

The online version contains supplementary material available at 10.1186/s40249-026-01441-w.

## Background

Whipple’s disease (WD), caused by *Tropheryma whipplei*, is a chronic multisystem infectious disorder characterized by heterogeneous clinical manifestations [1]. Although *T. whipplei* is ubiquitous in the environment—it is detected in wastewater treatment plant effluents, healthy carriers, and self-limiting gastrointestinal infections—WD itself remains exceedingly rare [2–5]. The classic form of WD predominantly affects Caucasians, non-Hispanic individuals, and those aged over 65 years, whereas it is exceptionally uncommon in Asian populations and nearly unreported in Africa [5–9]. Recent studies have reported an overall prevalence of 9.8 cases per million people in the United States, with no sex predilection [6]. The natural course of classic WD typically involves a prodromal phase manifesting as arthralgia, followed by a second stage dominated by gastrointestinal symptoms such as diarrhoea, nausea, abdominal pain, and weight loss. Rarely, the disease may involve the brain, heart, or lungs [10]. Owing to its nonspecific and variable presentations, delayed diagnosis and treatment often lead to high mortality [11].

Most patients exhibit proximal intestinal involvement, and duodenal biopsy with periodic acid-Schiff (PAS) staining remains a cornerstone diagnostic method, revealing foamy macrophages within the lamina propria. Polymerase chain reaction (PCR) targeting specific bacterial sequences plays a critical role in diagnosis, particularly for atypical cases or nonintestinal specimens [10]. In this case report, we present a Chinese patient with WD manifesting as multiple serous effusions, highlighting the significant diagnostic complexity. Definitive diagnosis requires histopathological examination, PAS staining, and PCR. This study aims to enhance the understanding of WD by exploring its rare clinical presentations, diagnostic challenges, and therapeutic dilemmas, thereby providing clinicians with valuable insights for improved management.

## Case presentation

### Multiple serous effusions

**Fig. 1:**
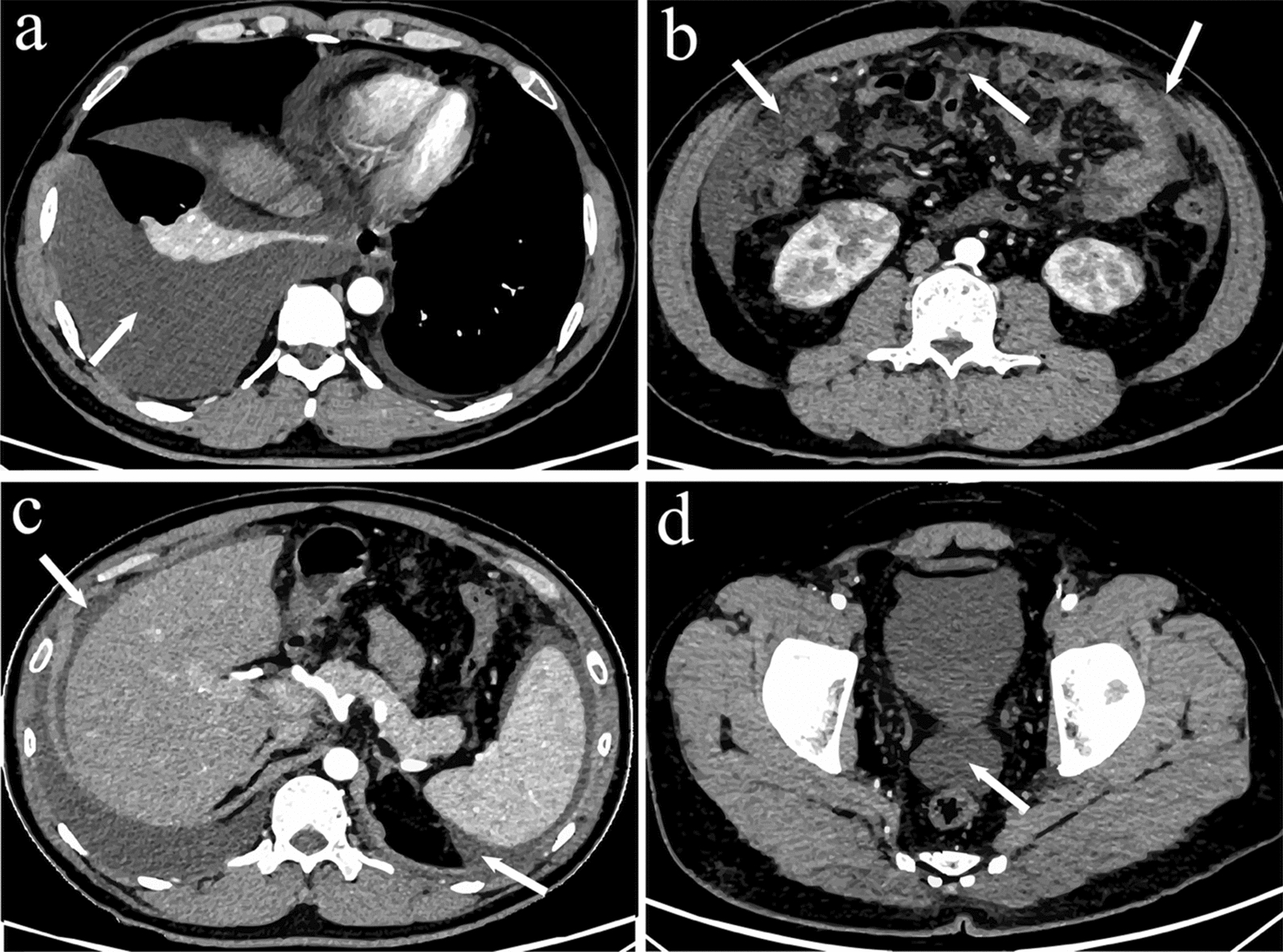
Contrast-enhanced Computed Tomography (CT) images demonstrating radiographic findings across different anatomical regions. **a** No abnormal parenchymal enhancement in the lungs. Bilateral pleural effusions (predominantly right‑sided) were accompanied by partial atelectasis of the right lower lobe. Minimal pericardial effusion (indicated by arrows). **b** Thickening of omental and mesenteric fat density, particularly in the omentum, displaying a "dirty" infiltrative appearance (indicated by arrows). **c** Fluid-density shadows along the hepatic and splenic surfaces (indicated by arrows). **d** Pelvic fluid collection (indicated by arrows)

On July 16, 2022, a 34-year-old man was admitted to our hospital with a 5-day history of recurrent fever, vomiting, diarrhoea, mild dry cough, and anorexia. Physical examination revealed normal mental status without altered consciousness or cognitive impairment. Palpable cervical lymphadenopathy was noted, but tonsillar enlargement was absent. Bilateral coarse breath sounds were observed, with diminished breath sounds over the right lung. No abnormal cardiac murmurs were detected. The abdomen was soft, non-tender, and had no rebound tenderness or palpable masses. Laboratory findings revealed pancytopenia, characterized by a white blood cell (WBC) count of 1.8 × 10⁹/L (reference range 3.5–9.5 × 10⁹/L), a neutrophil (NEUT) count of 1.25 × 10⁹/L (reference range 1.8–6.3 × 10⁹/L), and a lymphocyte (LYM) count of 0.36 × 10⁹/L (reference range 1.1–3.2 × 10⁹/L), along with thrombocytopenia (platelet (PLT) count: 80 × 10⁹/L; reference range 125–350 × 10⁹/L). Concurrently, the C-reactive protein (CRP) concentration was markedly elevated at 41.3 mg/L (reference range 0–10 mg/L), indicating a robust inflammatory response. Liver function tests revealed significant hepatocellular injury and cholestasis, with elevated concentrations of alanine aminotransferase (ALT), 229 U/L (reference range 9–50 U/L); aspartate aminotransferase (AST), 48 U/L (reference range 15–40 U/L); gamma-glutamyl transferase (GGT), 818 U/L (reference range 10–60 U/L); and alkaline phosphatase (ALP), 671 U/L (reference range 45–125 U/L). Additionally, hypoalbuminaemia was noted, with an albumin (ALB) concentration of 29.3 g/L (reference range 35–50 g/L). Collectively, these results suggested a systemic inflammatory state associated with impaired hepatic synthetic function, possibly attributed to either infectious or noninfectious aetiologies. Cervical lymph node ultrasound revealed hypoechoic nodules in bilateral level IV cervical regions (left: 0.8 × 0.5 cm; right: 0.7 × 0.6 cm) with well-defined margins and indistinct corticomedullary differentiation, suggesting reactive hyperplasia. Abdominal computed tomography (CT) revealed ascites, pelvic effusion, and bilateral pleural effusion incidentally identified during the abdominal scan, as the lower thorax was included in the imaging protocol. Contrast-enhanced CT further revealed abnormal opacity in both lungs (Fig S1). Bilateral pleural effusions (predominantly right‑sided) were accompanied by partial atelectasis of the right lower lobe, pericardial effusion, intra‑abdominal fluid collection, omental changes, and pelvic fluid collection (Fig. 1). Collectively, these findings raised a high suspicion of systemic vasculitis.

### Differential diagnosis

**Fig. 2:**
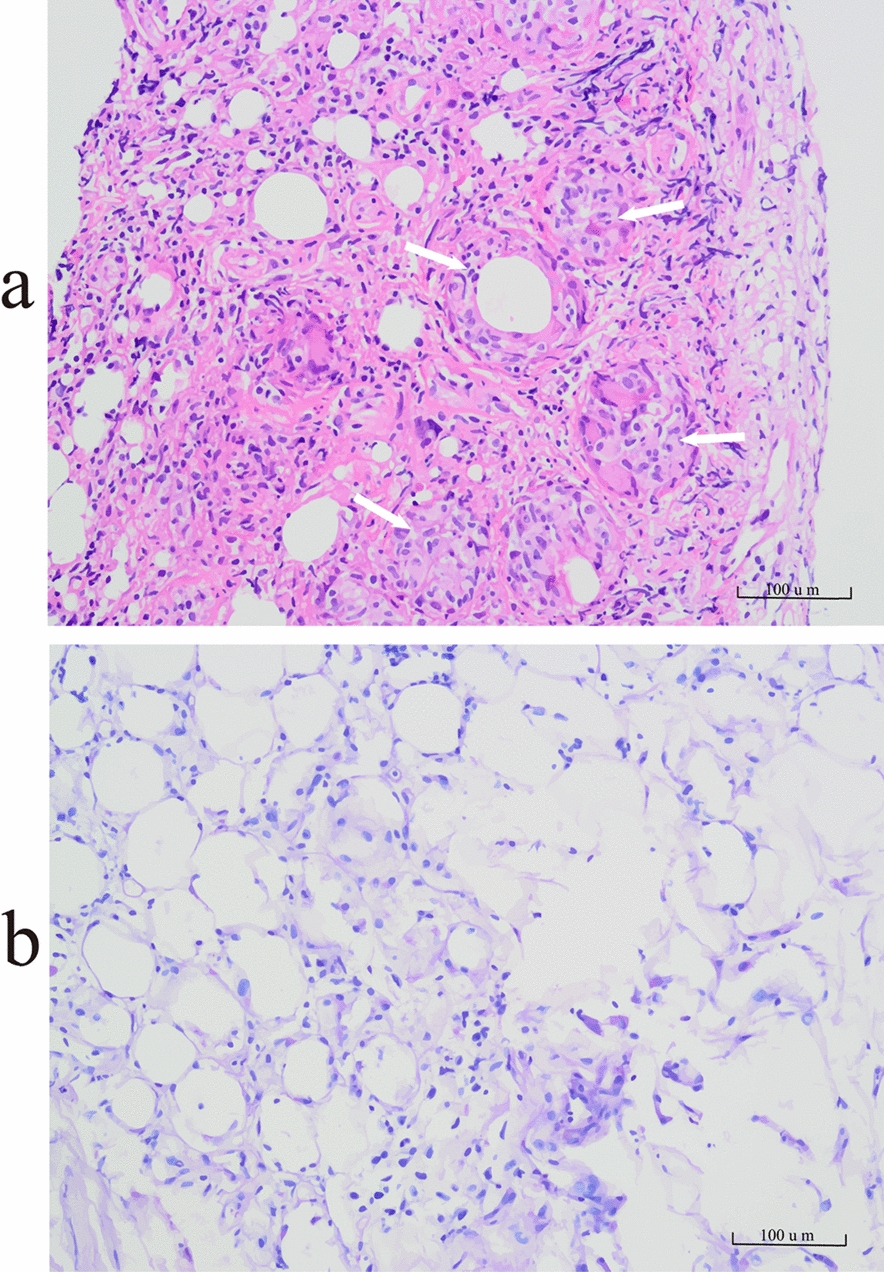
Histopathological findings of thoracoscopic pleural biopsy. **a** Hematoxylin and eosin staining demonstrates extensive adipose tissue with granuloma formation and marked lymphocytic infiltration (indicated by arrows, 20 × objective lens). **b** Periodic acid-Schiff staining reveals no intracytoplasmic granular positivity within macrophages (20 × objective lens)

The patient presented with fever, diarrhoea, and multiple serous effusions, raising initial suspicion of systemic vasculitis. Further testing revealed negative results for ANCA (c-ANCA/p-ANCA), ANA, anti-dsDNA, anti-Sm antibodies, anti-CCP antibodies, complement levels (C3/C4), and rheumatoid factor, ruling out systemic vasculitis. Analysis of the pleural fluid from right-sided thoracentesis revealed exudative characteristics (yellow turbid fluid with a specific gravity of 1.02, total protein concentration of 44 g/L, lactate dehydrogenase concentration of 1,000 U/L, adenosine deaminase (ADA) concentration of 64.6 U/L, glucose concentration of 6.58 mmol/L, and chloride concentration of 101 mmol/L). The fluid was predominantly mononuclear (WBC count 1,079 × 10^6^/L; mononuclear cells 83%, polymorphonuclear cells 17%). Elevated ADA levels and a mononuclear predominance suggested tuberculous pleuritis, although pleural malignancy remained a consideration. However, pleural fluid smears revealed no bacteria, fungi, or acid-fast bacilli; blood and pleural fluid cultures were negative; and pleural fluid cytology revealed no malignant cells. PCR assays for 13 pathogenic microorganisms targeting *Streptococcus pneumoniae*, *Staphylococcus aureus*, the *mecA* gene, *Escherichia coli*, *Klebsiella pneumoniae*, *Pseudomonas aeruginosa*, *Acinetobacter baumannii*, *Serratia marcescens*, *Haemophilus influenzae*, *Legionella pneumophila*, *Mycobacterium tuberculosis*, *Mycoplasma pneumoniae* and *Chlamydia pneumoniae* were performed on pleural fluid specimens. All the PCR assays yielded negative results. Blood tests, including T-SPOT. TB, CEA, CA125, CA199, and AFP levels were unremarkable. Empirical therapy with antibiotics (cefminox and piperacillin-tazobactam) (Table S1), closed thoracic drainage, hepatoprotective agents, probiotics, and antidiarrhoeals led to partial improvement in diarrhoea but persistent intermittent fever. A 10-day diagnostic trial of quadruple antitubercular therapy (isoniazid 300 mg/day, rifampin 600 mg/day, pyrazinamide 1,800 mg/day, and ethambutol 1,500 mg/day) was initiated but failed to resolve the fever. Persistent leukopenia and daily pleural effusion accumulation (300–500 ml/day) prompted further evaluation. Thoracoscopic pleural biopsy revealed lymphocyte and histocyte infiltration within adipose tissue, accompanied by fat necrosis and granuloma formation. Specific stains included PAS (−) (Fig. 2), acid-fast staining for TB (−), and Grocott’s methenamine silver (GMS) staining (−) (Fig S2). T-cell receptor gene rearrangement analysis revealed polyclonality, excluding tuberculosis, lymphoma, and pleural malignancy. Unstained paraffin sections were referred to Beijing Tiantan Hospital for consultation. The subsequent restaining results indicated that WD could not be excluded on the basis of morphological features. The immunohistochemical findings were as follows: CD163 (+) and CD68 (+); the specific staining results were as follows: PAS (+/−), acid-fast staining for TB (−), and weak acid-fast staining (−). The remaining pleural biopsy specimens were subsequently submitted for PCR testing, which could not be completed because of insufficient nucleic acid yield and the absence of an amplification curve.

On the basis of the suspected diagnosis of WD, the anti-infective regimen was adjusted to ceftriaxone (2 g, once daily). Given the prominent inflammatory exudation in the patient’s multiple serous cavities, methylprednisolone (MP, 40 mg/day) was added as combination therapy. After 3 days of antimicrobial therapy combined with glucocorticoid anti-inflammatory treatment, the patient’s body temperature returned to normal, and the drainage volume of the pleural effusion decreased significantly (10–20 ml per day). A recheck revealed that the CRP level had decreased to within the normal range, with an ALT concentration of 77 U/L (reference range 9–50 U/L) and an AST concentration within the normal range. At this point, the patient requested voluntary discharge for further consolidation therapy at a community medical institution. We recommended that the patient continue intravenous infusion of ceftriaxone (2 g, once daily) at a community hospital for a total course of 14 days, followed by sequential oral administration of trimethoprim-sulfamethoxazole (TMP-SMX, 160/800 mg, twice daily). After discharge, the patient was advised to continue oral prednisone (35 mg/day), with the dose reduced by 10 mg per week for gradual tapering and discontinuation.

### Disease recurrence

During sequential oral administration of TMP-SMX, the patient developed severe gastrointestinal reactions, manifested as frequent vomiting and anorexia. The symptoms were slightly relieved after adjusting the TMP-SMX dosage to 240/1,200 mg once daily and administering a symptomatic treatment for acid suppression and gastric mucosa protection. 1 week after the gradual tapering and discontinuation of glucocorticoids, the patient developed recurrent fever, with a maximum body temperature of 39.5 °C, which was accompanied by chills, rigor, nausea, acid regurgitation and vomiting. After receiving empirical anti-infective treatment with ceftriaxone for 4 days and intermittent anti-inflammatory treatment with dexamethasone 2 times in a community clinic (Table S1), the patient’s fever improved, but acid regurgitation, nausea and vomiting remained prominent; thus, the patient was readmitted to our hospital. A retrospective review of the medical history revealed that the patient had experienced a body weight loss of 15 kg since the onset of the disease.

A complete set of laboratory examinations was performed after admission. Routine blood tests revealed a WBC count of 2.73 × 10^9^/L (reference range 3.5–9.5 × 10^9^/L), a NEUT count of 1.77 × 10^9^/L (reference range 1.8–6.3 × 10^9^/L), a LYM count of 0.68 × 10^9^/L (reference range 1.1–3.2 × 10^9^/L), a PLT count of 110 × 10^9^/L (reference range 125–350 × 10^9^/L), and a CRP concentration of 36.41 mg/L (reference range 0–10 mg/L). Liver function tests revealed an ALT concentration of 330 U/L (reference range 9–50 U/L), an AST concentration of 641 U/L (reference range: 15–40 U/L), a GGT concentration of 283 U/L (reference range 10–60 U/L), an ALP concentration of 282 U/L (reference range 45–125 U/L), and an ALB concentration of 31 g/L (reference range: 35–50 g/L). The serum ferritin concentration was 5,238 ng/ml (reference range 13–400 ng/ml). The absolute CD4^+^ T-cell count was 151/μl (reference range 441–2,156/μl) No pathogenic bacteria were detected in bilateral double-bottle blood cultures. CT revealed inflammation in the bilateral lower lobes of the lungs, recurrent bilateral pleural effusion, and complications of abdominopelvic effusion and abdominopelvic peritoneal thickening (Fig S3).

**Fig. 3:**
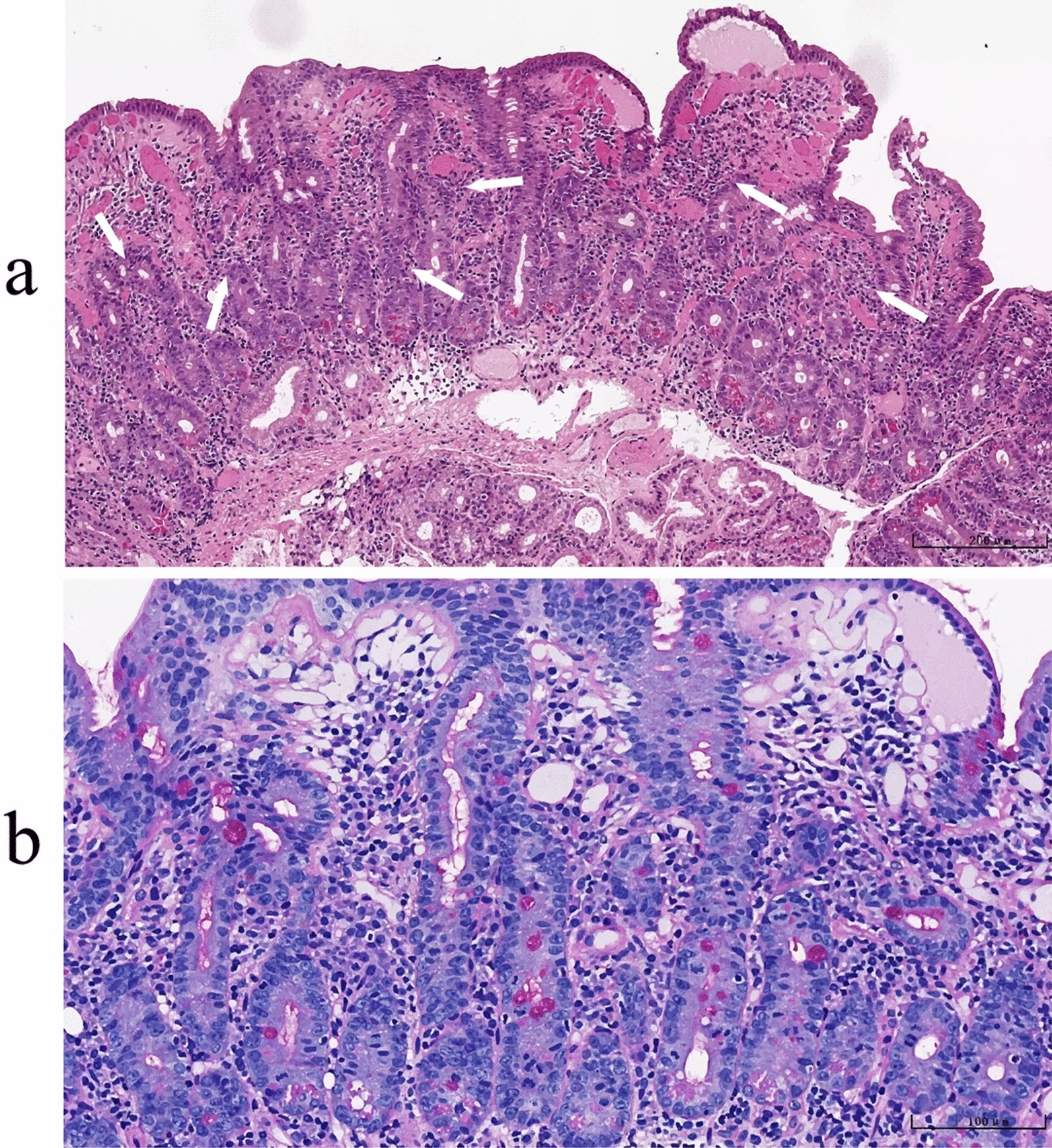
Histopathology of duodenal transitional zone. **a** Hematoxylin and eosin stained tissue section shows chronic active inflammation of mucosa with significant erosion, congestion and edema in the lamina propria accompanied by infiltration of numerous lymphocytes, plasma cells and neutrophils (indicated by arrows, 10 × objective lens). **b** Periodic acid-Schiff staining showed no positive expression. The specimen was photographed using a 20 × objective lens

A comprehensive analysis of the above examination results temporarily excluded newly developed opportunistic infections. The recurrent fever in the patient might have been related to poor control of the primary disease because of poor tolerance of TMP-SMX and was likely attributable to immune reconstitution inflammatory syndrome (IRIS). In addition, the certainty of the diagnosis of WD needed to be reevaluated. Given the prominent gastrointestinal symptoms and severe body weight loss in the patient, further gastroenteroscopy was performed to confirm the diagnosis. Histopathological findings revealed chronic active inflammation with prominent erosions in the duodenal transitional mucosa. The lamina propria exhibited congestion, oedema, and dense infiltration of lymphocytes, plasma cells, and neutrophils. PAS staining was negative (Fig. 3). Similar histopathological features were observed in the descending duodenum (Fig S4). PCR analysis of paraffin-embedded duodenal tissue was negative for *T. whipplei* DNA. Owing to the persistent fever and a continuous increase in pleural effusion volume, treatment with MP (40 mg/day) was reinitiated to control systemic inflammation and fever.

### Confirmation of the diagnosis

**Fig. 4:**
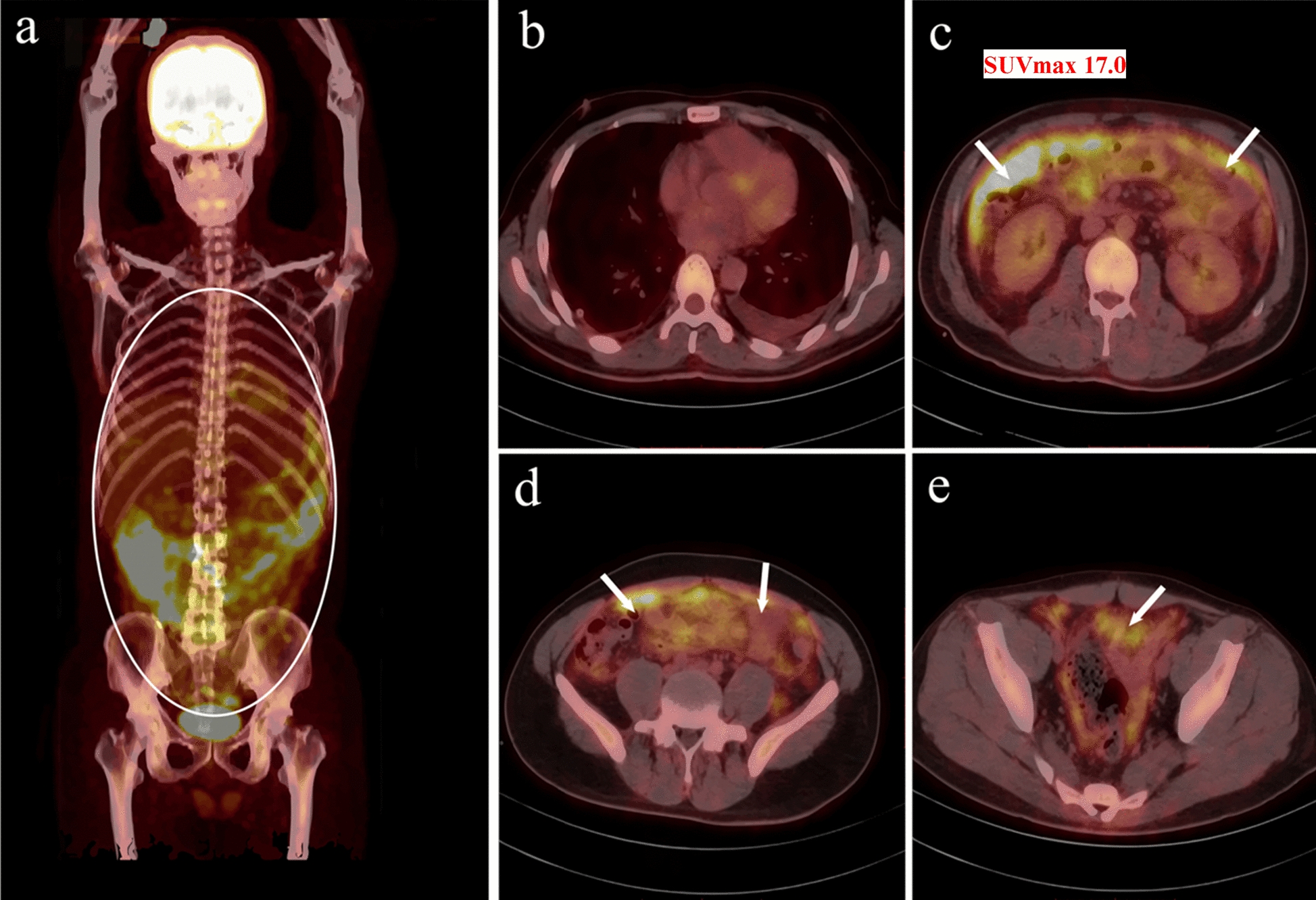
18F-fluorodeoxyglucose positron emission tomography (18F-FDG PET) demonstrating differential tracer uptake across multiple lesions. **a** Yellow-highlighted area reveals intense focal 18F-FDG uptake in local tissue (lesion marked with a circle). **b** No abnormal FDG metabolic activity is observed in the chest. **c**, **d**, **e** Marked hypermetabolic foci are noted in the peritoneal region (predominantly involving the greater omentum) and pelvis, with a maximum standardized uptake value (SUVmax) of 17.0 (indicated by arrows)

**Fig. 5:**
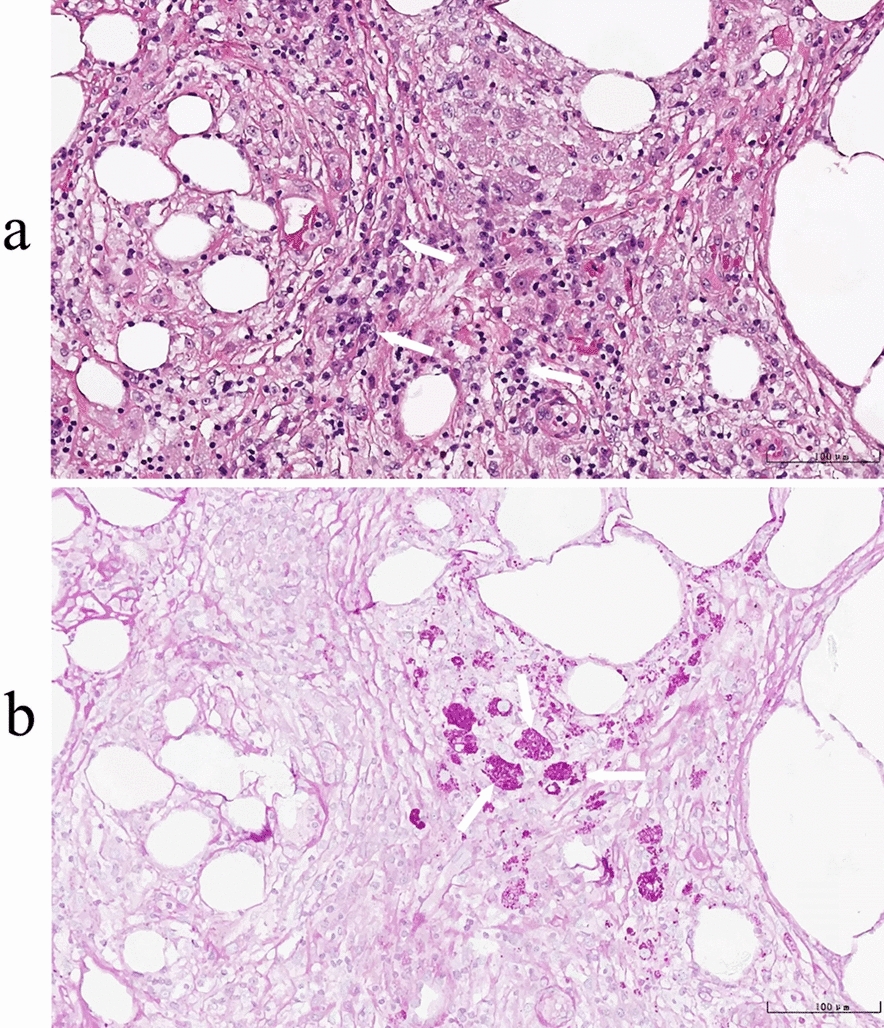
Histopathology of the greater omentum. **a** Hematoxylin and eosin staining: chronic inflammation with fat necrosis is observed, featuring abundant infiltration of lymphocytes, plasma cells, and foamy macrophages. Localized granuloma-like structures are present (indicated by arrows, 20 × objective lens). **b** Periodic acid-Schiff (PAS) staining: Strongly PAS-positive macrophages are identified (indicated by arrows, 20 × objective lens)

Further 18F-fluorodeoxyglucose positron emission tomography (18F-FDG PET) revealed extensive peritoneal thickening in the abdominal and pelvic cavities with hypermetabolism (SUVmax 17.0), requiring differentiation between inflammatory granuloma and malignancy (Fig. 4). To determine the aetiology, a laparoscopic-assisted omental biopsy was performed. Histopathological examination revealed chronic inflammation with fat necrosis in the omentum, characterized by infiltration of abundant lymphocytes, plasma cells, and foamy macrophages, along with granuloma-like structures and vascular dilation/congestion. PAS staining was positive (Fig. 5). *T. whipplei* DNA was detected via PCR in paraffin-embedded tissue samples (Supplemental Data). At this point, the diagnosis of WD was confirmed in the patient, and the treatment regimen was adjusted accordingly: oral doxycycline (100 mg, twice daily) combined with hydroxychloroquine (200 mg, twice daily), supplemented with stepwise tapering of prednisone for anti-inflammatory therapy (dose tapering: 30 mg/day × 3 days → 20 mg/day × 3 days → 10 mg/day × 3 days → 5 mg/day × 7 days, and then discontinued). Prior to treatment, complete echocardiography and contrast-enhanced cranial magnetic resonance imaging were performed, and no cardiac or craniocerebral involvement was found. After the above treatment, the patient’s body temperature gradually returned to normal, the amount of pleural effusion decreased, and the patient was successfully discharged after sustained remission of the condition.

After discharge, the patient was given individualized follow-up, with preset time points at 1, 3, 6, 12, and 24 months post-discharge. Follow-up at 1 month: No fever or vomiting was noted; only mild nausea, no recurrence of pleural effusion, and an initial remissive condition were noted. Follow-up at 3 months: The condition remained stable, with increased body weight and no ocular or nervous system discomfort. An electrocardiogram (ECG) showed no QT interval prolongation; routine blood tests, the CRP level, and liver and kidney functions were normal. The absolute CD4^+^ T lymphocyte count was 253/µl (reference range: 441–2,156/μl), which was still lower than the normal level. Chest and abdominal CT revealed absorption of the left pleural effusion, a small amount of residual pleural effusion on the right side, and no significant changes in the manifestations of increased peritoneal fat stranding with exudation (Fig S5-1). Follow-up at 6 months: The patient did not return to the hospital as planned. Follow-up at 12 months: The absolute CD4^+^ T lymphocyte count had returned to normal, the bilateral pleural effusion was absorbed, and peritoneal exudation had significantly improved (Fig S5-2). An ECG showed no abnormalities, and fundus photography revealed no retinopathy (Fig S6). Discontinuation of hydroxychloroquine was recommended, but the patient continued to take the drug independently until 15 months post-discharge, with no adverse drug-related reactions observed during this period. Follow-up at 24 months: No significant changes were noted in peritoneal exudation compared with that at 12 months post-discharge (Fig S5-3). The patient took medications regularly as prescribed throughout the follow-up period, with no gastrointestinal discomfort; his physical strength and mental state were significantly improved. Currently, the patient continues to receive oral doxycycline (100 mg, twice daily) for maintenance treatment. Regular follow-up once a year thereafter was recommended to monitor disease changes and prevent recurrence.

## Discussion

Classic WD, caused by *T. whipplei* infection of intestinal macrophages, presents with chronic gastrointestinal symptoms and multisystem involvement [12–14]. Chronic localized infections without classical gastrointestinal manifestations are being increasingly recognized and often manifest as chronic arthritis, endocarditis, or central nervous system disease [7]. Studies suggest that nonspecific symptoms such as fever, lymphadenopathy, and pancytopenia should raise suspicion for WD, in addition to lymphoma or granulomatous reticuloendothelial disorders [15–17]. Owing to the rarity and nonspecific clinical features of WD, its diagnosis remains challenging [18]. Classic WD is confirmed by the presence of foamy, PAS-positive macrophages in affected tissues (typically the small intestine) with negative acid-fast staining [11]. PCR testing for *T. whipplei* has become increasingly available, significantly aiding in disease confirmation [19, 20]. This case, characterized by multiple serous effusions and diffuse peritoneal thickening with hypermetabolism, underscores the diagnostic complexity of WD and contributes to the clinical understanding of atypical WD presentations. Through extensive exclusion of systemic vasculitis, tuberculous pleuritis, and malignancy via serological, imaging, and histopathological evaluations, this report indicated that further investigation for specific pathogenic infections was warranted. A definitive diagnosis of WD was ultimately confirmed by omental pathological examination, PAS-specific staining and PCR assay in combination.

WD can be fatal if not treated with appropriate antibiotics. A single-centre randomized controlled trial in Germany recommended initial treatment with ceftriaxone (2 g once daily) or meropenem (1 g three times daily) for 2 weeks, followed by oral TMP-SMX for 12 months [21]. In vivo evidence for ceftriaxone–TMP‑SMX treatment remains limited [21–23]. Doxycycline plus hydroxychloroquine is the only regimen with proven in vitro bactericidal activity [23–25]. A 2025 study published in the Lancet Infectious Diseases demonstrated its safety and non‑inferiority compared with those of ceftriaxone–TMP‑SMX [26]. In the present case, the patient experienced disease recurrence after corticosteroid discontinuation. Re-examination revealed elevated levels of CRP, transaminases and ferritin and a reduced absolute CD4^+^ T-cell count, and imaging examinations revealed recurrent pleural effusion. On the basis of the patient’s clinical manifestations, we speculated that the recurrent fever was most likely attributable to IRIS. After the therapeutic regimen was adjusted to doxycycline combined with hydroxychloroquine, corticosteroid therapy was promptly initiated for the patient because of a high clinical suspicion of IRIS, leading to sustained remission of the patient’s condition.

IRIS was first defined by Shelburne et al. in 2002 as paradoxical symptom exacerbation following immune recovery [27, 28]. WD patients frequently exhibit immunosuppression with a reduced CD4^+^ T-cell count, a recognized IRIS risk factor [1, 29]. Diagnosis relies on clinical criteria: initial improvement within 3 weeks, recurrent inflammation > 1 week after excluding nosocomial causes, and negative histopathology/PCR results ruling out recurrence [30]. IRIS may overlap with early WD recovery, complicating the determinations of its onset time and disease course [21, 30]. No proven IRIS therapy exists; corticosteroids have been used empirically [31, 32], and thalidomide has shown success in case series [33, 34]. This case suggests that in clinical practice, IRIS should be considered in all WD patients who develop unexplained fever or other inflammatory symptoms after the initiation of treatment, and targeted diagnostic and therapeutic measures should be taken in a timely manner.

Several limitations exist. First, a baseline ophthalmic examination was not performed before the initiation of hydroxychloroquine, and routine fundoscopy was not conducted during follow‑up. Second, therapeutic drug monitoring was unavailable because of laboratory constraints. Third, given the invasiveness of laparoscopy, follow‑up re‑evaluations of omental histopathology and *T. whipplei* PCR were not performed.

## Conclusion

*T. whipplei* infection presents with diverse clinical subtypes and lacks specific symptoms, making diagnosis challenging. Clinicians should consider *T. whipplei* infection in patients with various clinical manifestations. Multiple serous cavity effusions are relatively rare in WD patients, and early recognition and prompt treatment of *T. whipplei* infection are critical for patient prognosis. IRIS should be considered in all WD patients who develop unexplained fever or other inflammatory symptoms after the initiation of treatment. This case highlights the nonspecific clinical features and diagnostic challenges of WD, contributing to the clinical diagnostic understanding of this condition.

## Supplementary Information

Supplementary Material 1.

Supplementary Material 2.

## Data Availability

The data associated with this study are included and presented in this article. Additional materials are available from the corresponding author upon reasonable request.

## Abbreviations

WD: Whipple’s disease
PCR: Polymerase chain reaction
PAS: Periodic acid–schiff
WBC: White blood cell
NEUT: Neutrophil
LYM: Lymphocyte
PLT: Platelet
CRP: C-reactive protein
ALT: Alanine aminotransferase
AST: Aspartate aminotransferase
GGT: Gamma-gluttonyl transferase
ALP: Alkaline phosphatase
ALB: Albumin
ADA: Adenine dreaminess
CT: Computed tomography
GMS: Grocott’s methenamine silver
MP: Methylprednisolone
TMP-SMX: Trimethoprim-sulfamethoxazole
IRIS: Immune reconstitution inflammatory syndrome
H&E: Hematoxylin and eosin
18F-FDG PET: 18F-Fluorodeoxyglucose positron emission tomography
ECG: Electrocardiogram
