# Supplementary material for: Whipple’s disease with multiple serous effusions as the clinical manifestation: a case report and literature review
Source: Infect Dis Poverty. 2026 Apr 13;15:43. doi: 10.1186/s40249-026-01441-w (PMC13072459; doi:10.1186/s40249-026-01441-w)
Supplement: Supplementary file 1 — Supplementary Material 1. [file 40249_2026_1441_MOESM1_ESM.docx]

**Supplemental data**

**Sample Information**

Sample type: Biopsy sample

Receipt day: November 8, 2022

Report day: November 10, 2022

Polymerase chain reaction (PCR) was carried out to amplify a partial sequence of *Tropheryma whipplei*, and first - generation sequencing technology was used for sequencing.

1. Total DNA was extracted from the patient's paraffin-embedded tissues using the QIAamp DNA Blood Mini Kit (Qiagen) according to the manufacturer's instructions.
2. The PCR was performed with TB Green Premix Ex Taq according to the manufacturer’s instructions (Takara, Dalian, Liaoning) on an ABI QuantStudio5 System (Applied Biosystems).

The sequences of primers were as follows:

Forward primer:5’-CGCCTGTGTGCCAATGTAGA-3’;

Reverse primer:5’-ACTGATATACACGAGCGGCAT-3.

c. The ITS region (the non-coding region between the 16S - 23S rRNA genes) has a higher mutation rate than the 16S rDNA and stronger specificity. It is the gold-standard target for the diagnosis of Whipple's disease.

**Conclusion**

The 16S-23S ribosomal RNA spacer region (partial sequence: 144 bases) measured from the sample

1 TCGCCTGTGTGCCAATGTAGAACGGGT

28 GATCCTTGCGGTCGAGCTCAATAAATT

55 CAATCAGGTCTTGCTCTGTATTCCGCC

82 CAGAGACTATAAGCCCACGCTCAACCA

109 GTCGCTGTGCATAGCCATTATTTACTT

136 AAAAGGGGT

| 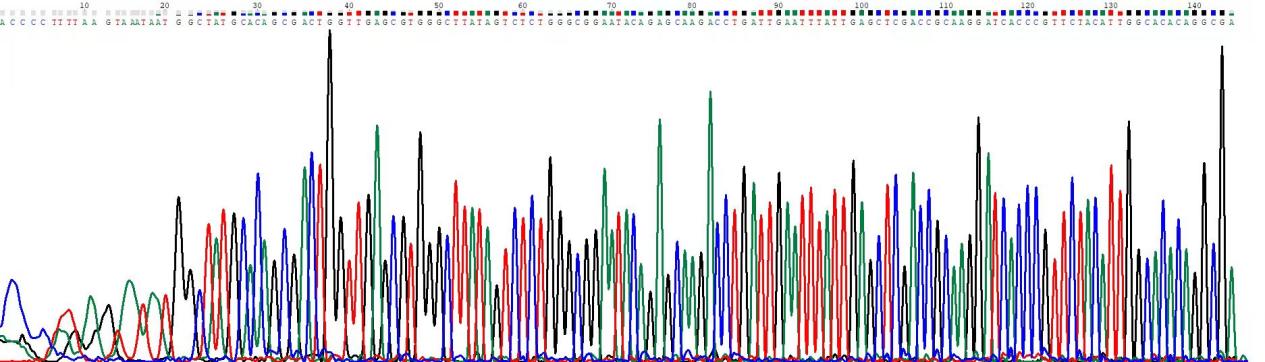 |
| --- |
| Fig S7 Nucleotide sequencing chromatogram |

| 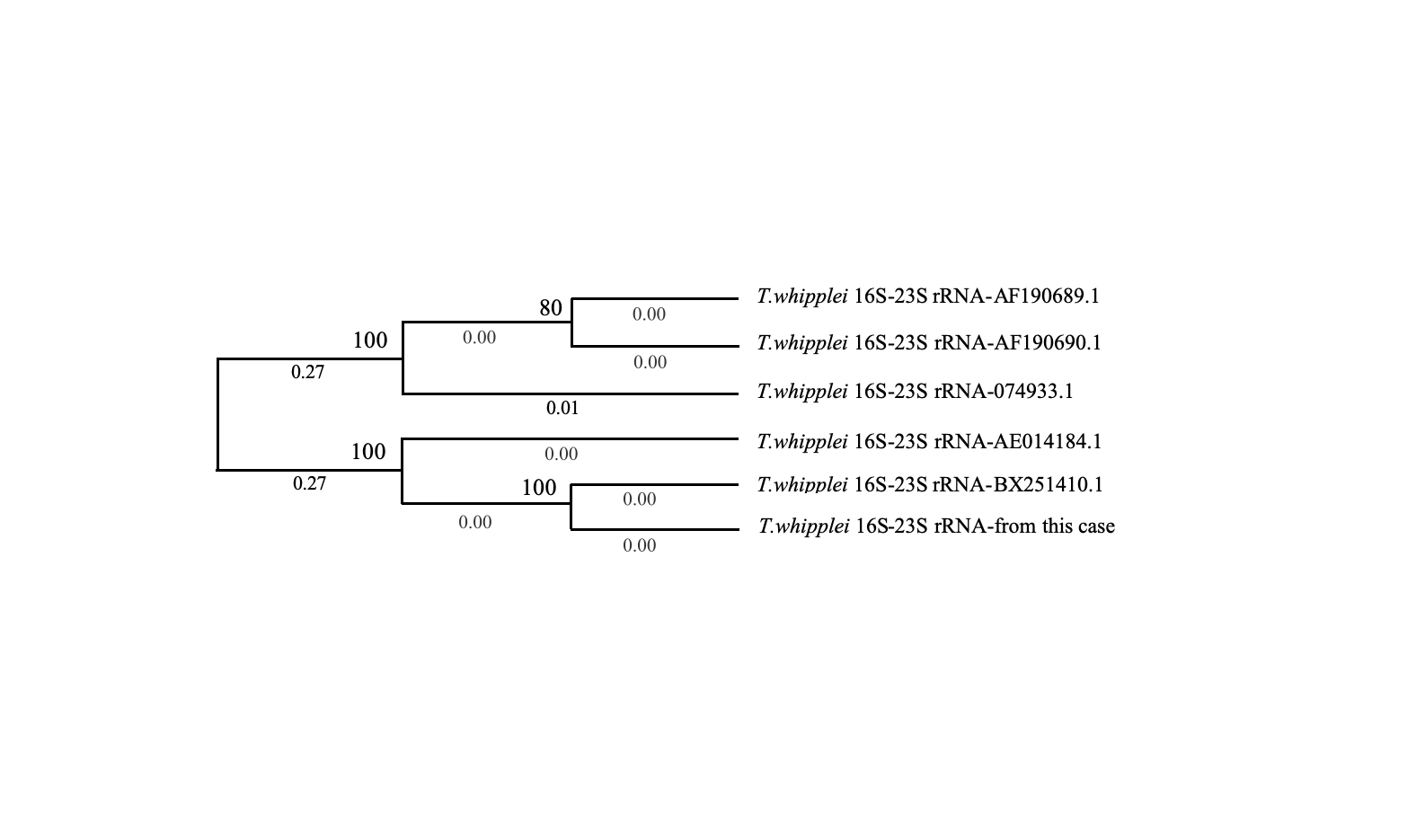 |
| --- |
| Fig S8 Phylogenetic tree of *T. whipplei* 16S -23S rRNA sequence. |

Nucleotide sequence alignment and MEGA phylogenetic tree results indicate that the obtained partial sequence is derived from *Tropheryma whipplei***.**

**Report Information**

Bacterium: *Tropheryma whipplei*
