# Supplementary material for: Whipple’s disease with multiple serous effusions as the clinical manifestation: a case report and literature review
Source: Infect Dis Poverty. 2026 Apr 13;15:43. doi: 10.1186/s40249-026-01441-w (PMC13072459; doi:10.1186/s40249-026-01441-w)
Supplement: Supplementary file 2 — Supplementary Material 2. [file 40249_2026_1441_MOESM2_ESM.docx]

**Supplemental** **Figures**

| **Fig S1** |
| --- |
| 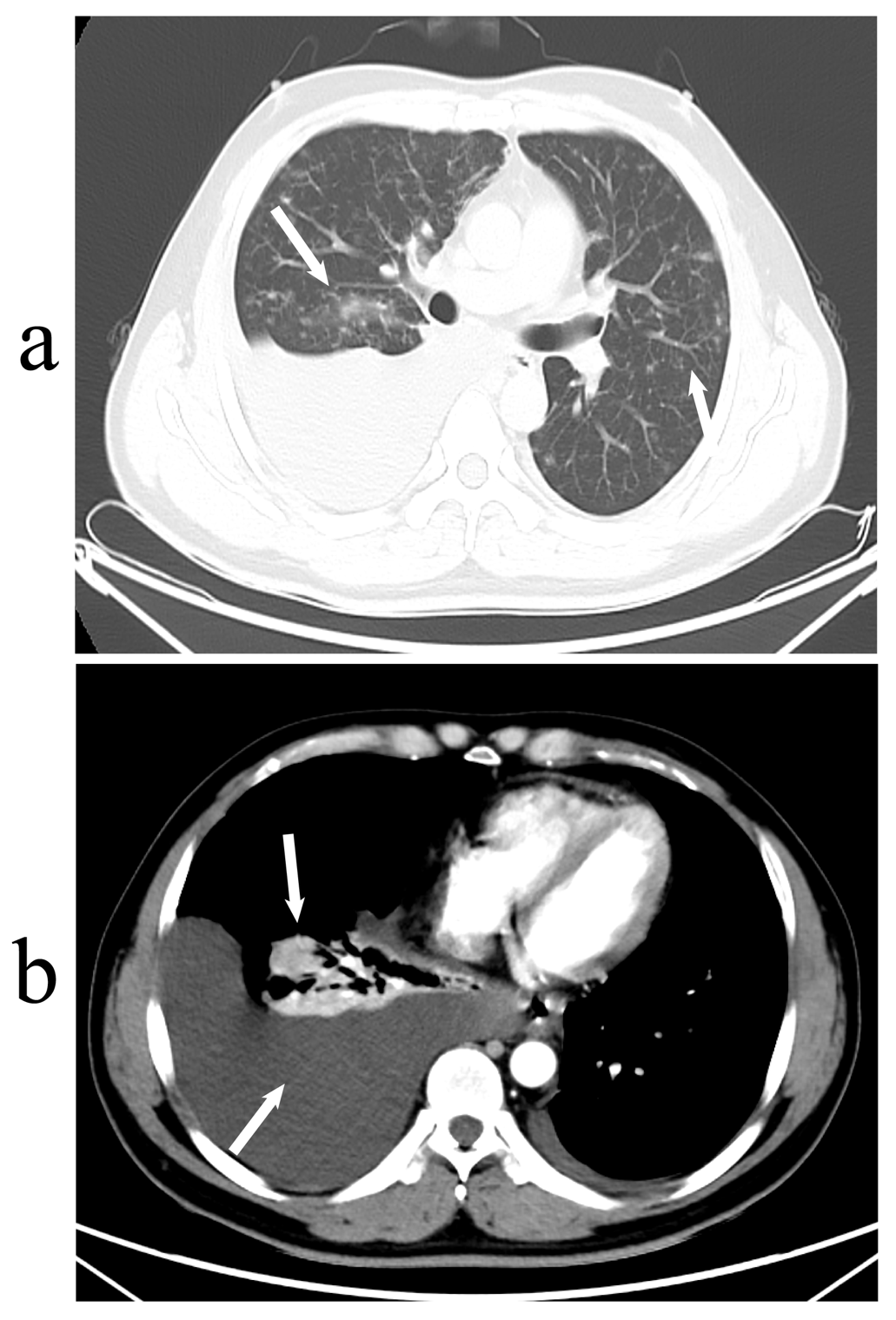 |
| Fig S1 Contrast-enhanced computed tomography (CT) images demonstrating radiographic findings in lung window and mediastinal window. a Lung window: inflammatory opacities are present in both lungs, accompanied by bilateral pleural effusions, which are more prominent on the right side (indicated by arrows). b Mediastinal window: no abnormal enhancement is observed; atelectasis is noted in the right lung (indicated by arrows). |

| **Fig S2** |
| --- |
| 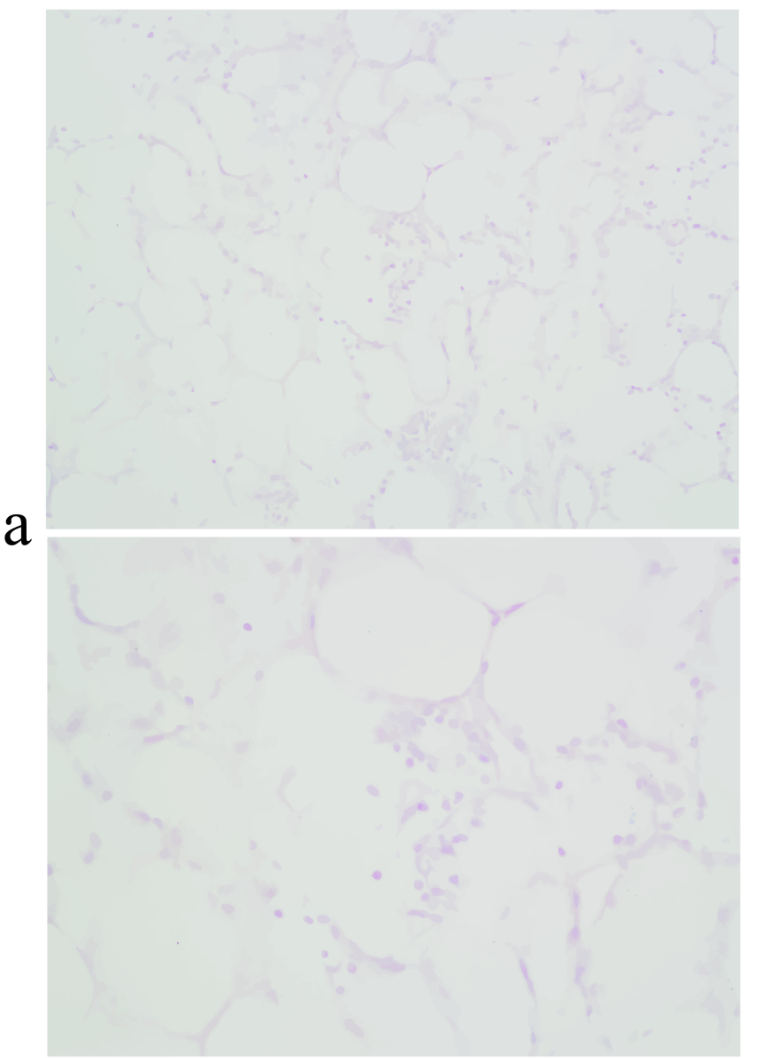  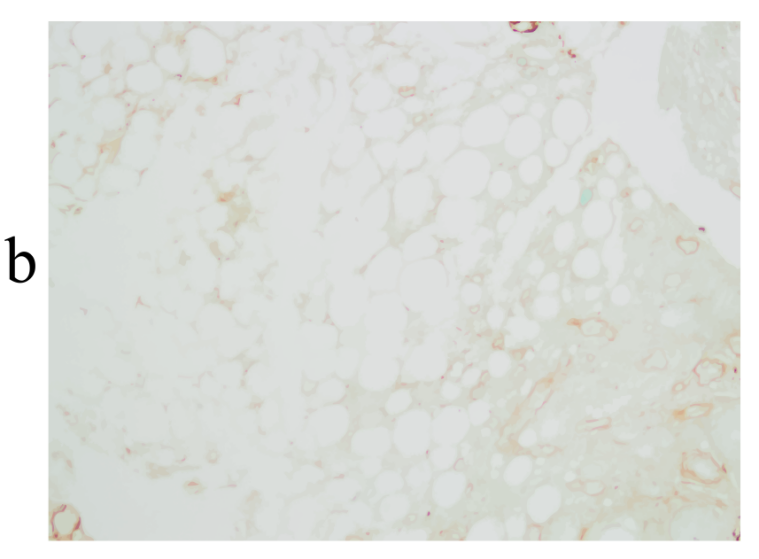 |
| Fig S2 Histopathological analysis of thoracoscopic pleural biopsy. a Acid-fast staining was negative. Specimen a (upper): 20× objective lens. Specimen a (lower): 40× objective lens. b Grocott’s methenamine silver (GMS) staining showed no fungal hyphae. Specimen b imaged with a 20× objective lens. |

| **Fig S3** | |
| --- | --- |
| 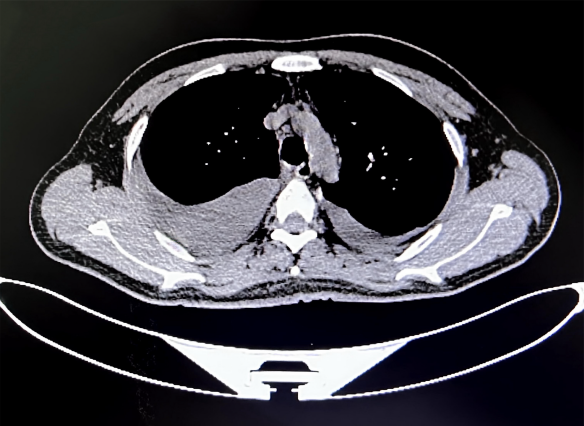  a | 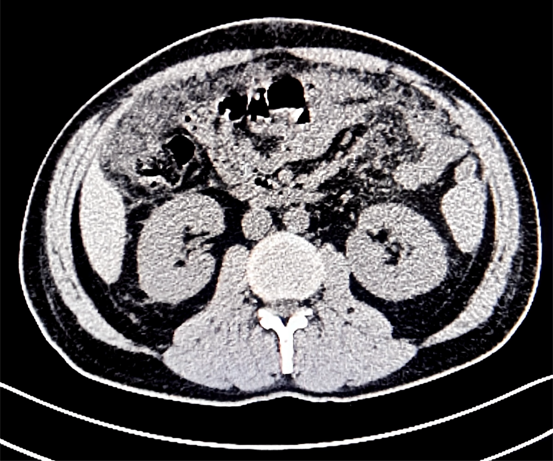  b |
| Fig S3 Computed tomography scans demonstrated the imaging findings corresponding to the patient's disease progression. a Recurrent pleural effusion, predominantly on the right side (indicated by arrows). b Peritoneal thickening and effusion (indicated by arrows). | |

| **Fig S4** |
| --- |
| 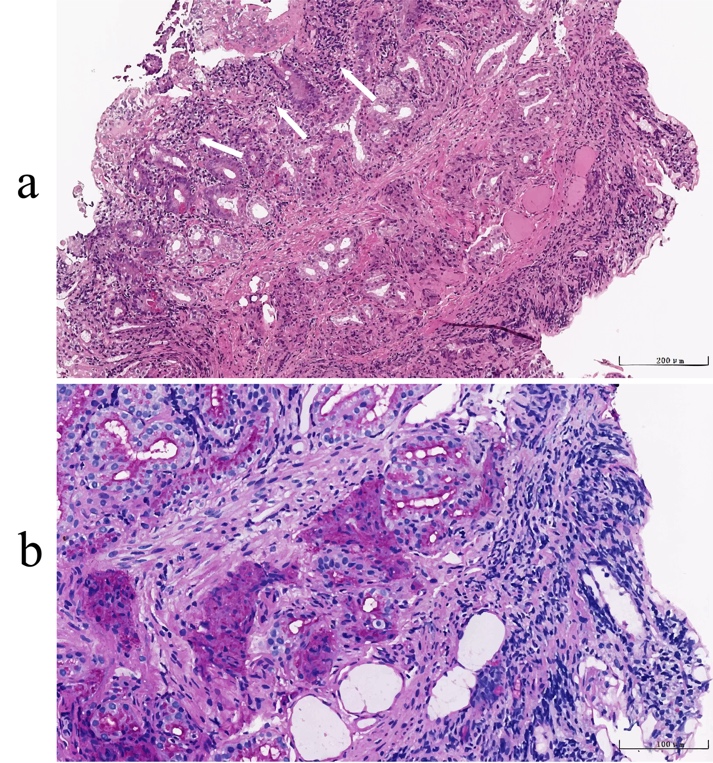 |
| Fig S4 Histopathological findings of the descending duodenum. a Hematoxylin and eosin (H&E) staining of the descending duodenum reveals chronic active inflammation with prominent mucosal erosions. The lamina propria shows congestion, edema, and dense infiltration of lymphocytes, plasma cells, and neutrophils (indicated by arrows, 10× objective lens). b Periodic acid-Schiff (PAS) staining demonstrates no positive staining (20× objective lens). |

| **Fig S5 Comparison of imaging changes during the follow-up period** | |
| --- | --- |
| 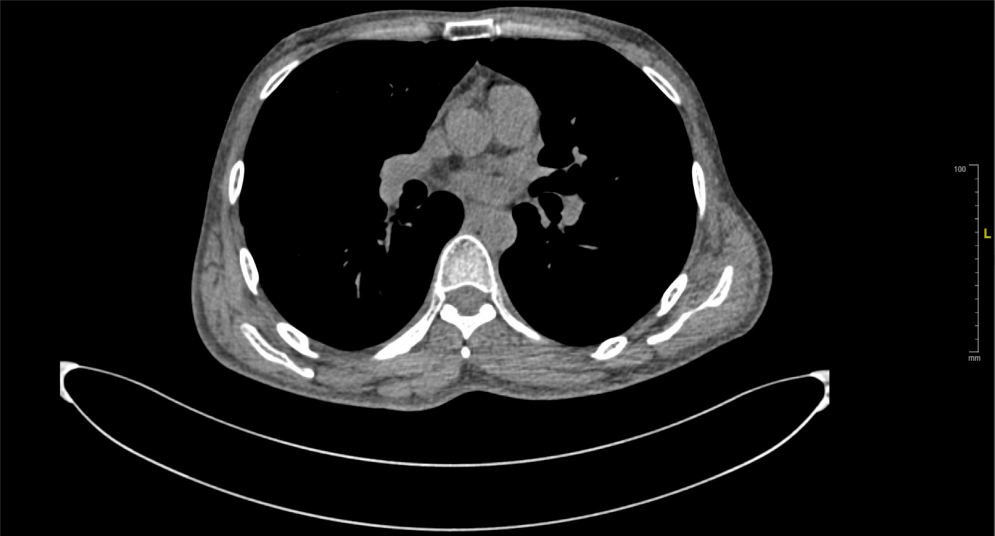 | 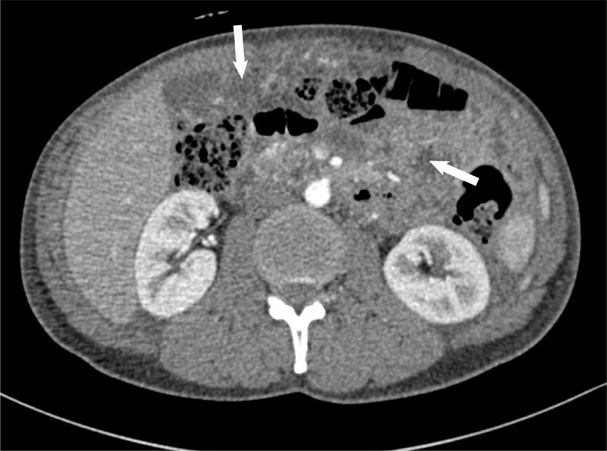 |
| Fig S5-1 Follow-up at 3 months, chest and abdominal computed tomography (CT) revealed complete absorption of left-sided pleural effusion, a small amount of residual right-sided pleural effusion (indicated by arrows), and manifestations of increased fat stranding with exudation that were roughly consistent with previous findings (indicated by arrows). | |
| 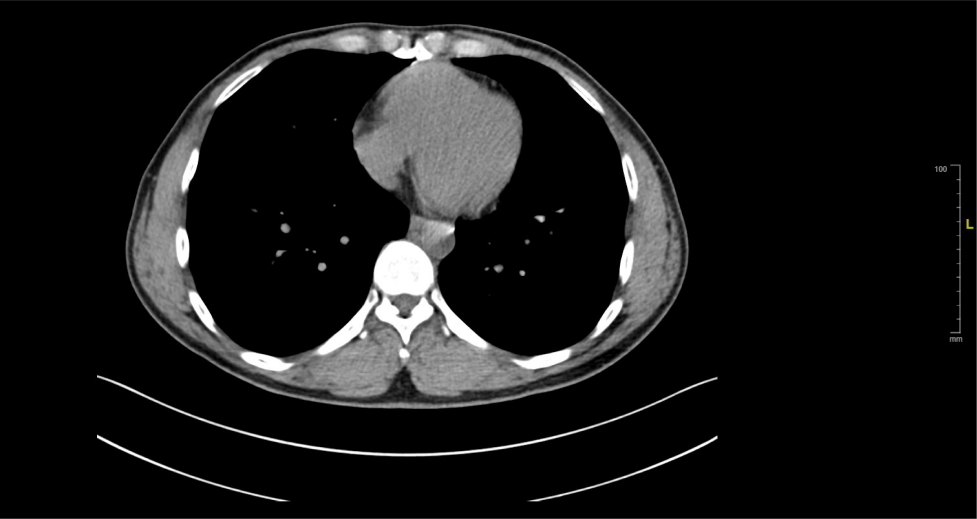 | 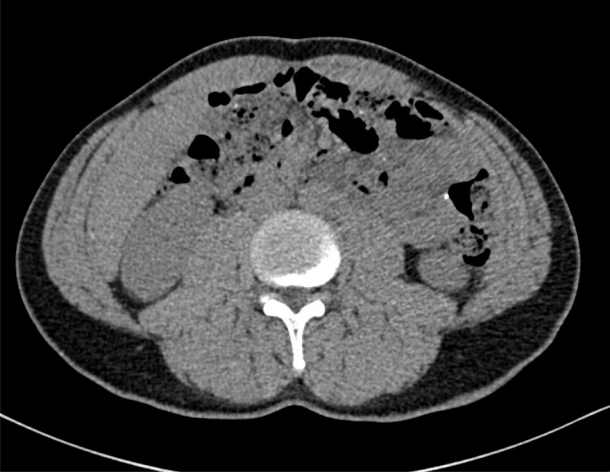 |
| Fig S5-2 Follow-up at 12 months, chest CT showed complete absorption of bilateral pleural effusion; abdominal CT indicated a significant improvement in the manifestations of increased fat stranding with exudation compared with prior results. | |
| 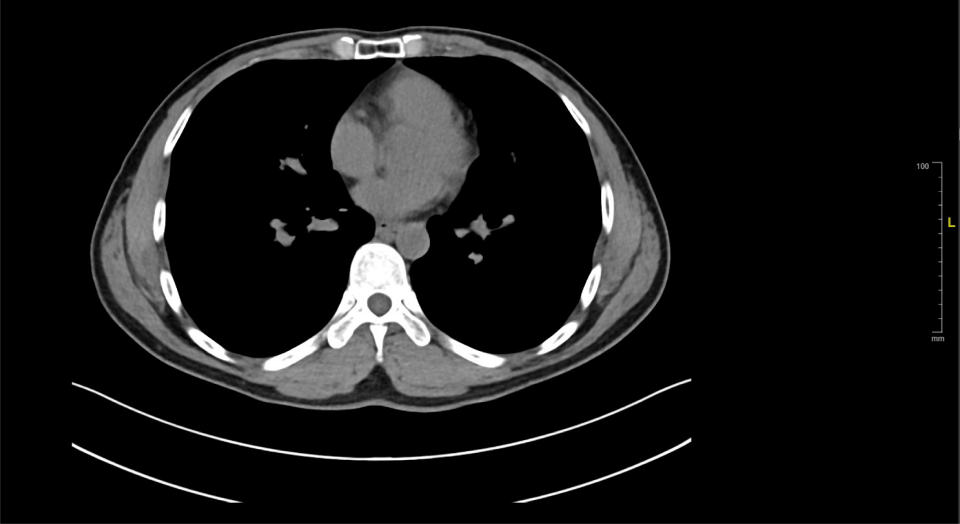 | 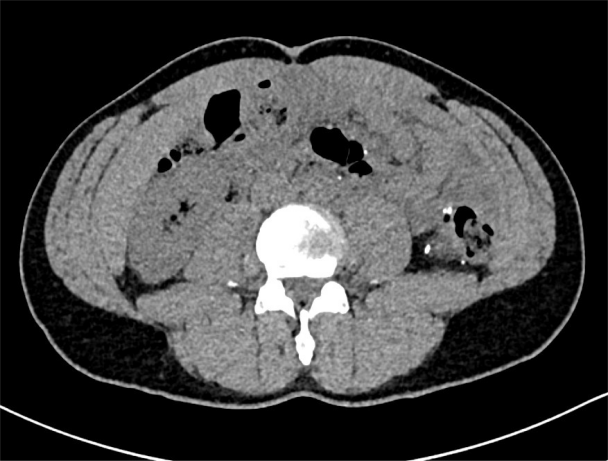 |
| Fig S5-3 Follow-up at 24 months, abdominal CT showed manifestations of increased fat stranding with exudation with no significant changes compared with the 12-month follow-up results. The patient's condition continued to improve. | |

| **Fig S6** **Fundus photography examination** | |
| --- | --- |
| 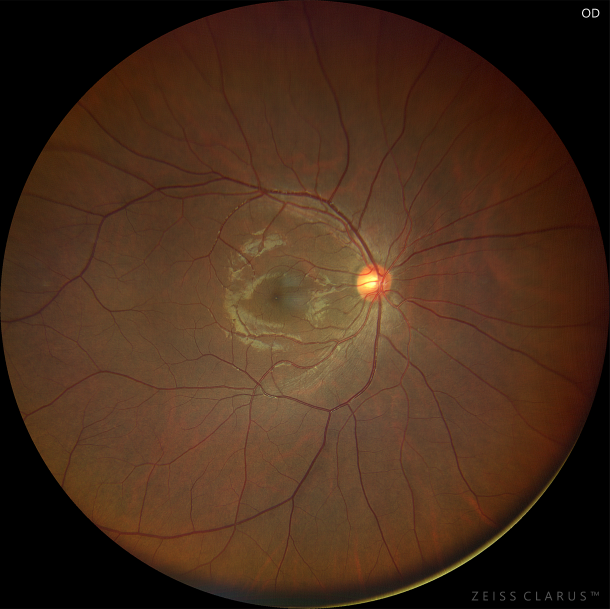 | 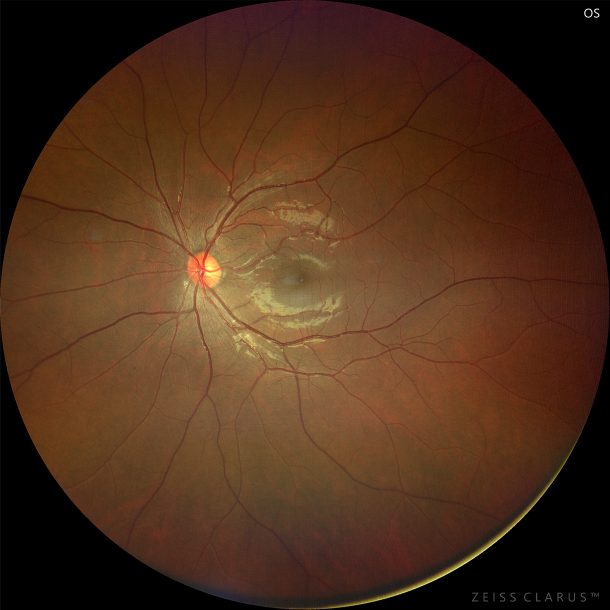 |
| Fig S6 Follow-up at 12 months, fundus photography revealed no retinopathy. | |

**Supplemental Tables**

| **Table S1** **Detailed medication regimens, dosages, and treatment courses administered to the patient.** | | | | |
| --- | --- | --- | --- | --- |
| **medication** | **dose** | **route** | **frequency** | **treatment duration** |
| cefminox | 2.0 g | intravenous drip | q12h | 5 days |
| piperacillin-tazobactam | 4.5 g | intravenous drip | q8h | 7 days |
| ceftriaxone | 2.0 g | intravenous drip | qd | 4 days |
| dexamethasone | 5.0 mg | intravenous bolus | interdose | two doses |

**Table S2** **Experimental reagents and devices used in the study, including respective manufacturers and models.**

| **Reagents** | **Manufacturers/ Models** |
| --- | --- |
| Routine blood test | Sysmex/XN-9000 |
| Liver function test | Roche, Switzerland/Cobas C 702 |
| Tumor marker test | Roche, Switzerland/Cobas e 801 |
| Autoantibody test | EUROIMMUN Medical Laboratory Diagnostics Co., Ltd., Hangzhou, China |
| Autoantibody test (anti-CCP) | Abbott Gmbh |
| 18F-fluorodeoxyglucose | Beijing Atom High Tech Co., Ltd., China |
| Hematoxylin staining | Wuxi Jiangyuan Industrial Technology & Trade Corporation, China |
| Hematoxylin and eosin (H&E) staining | China National Pharmaceutical Group Corporation, China |
| Grocott’s methenamine silver (GMS) staining | China National Pharmaceutical Group Corporation, China |
| Periodic acid-Schiff (PAS) staining | Fuzhou Maixin Biotech Co., Ltd., China |
| Acid-fast staining | Zhuhai Baso Biotechnology Co., Ltd., China |
| Immunohistochemistry (IHC) | Beijing Zhongshan Jinqiao Biotechnology Co., Ltd., China |
| Pathogen-specific PCR primers | Sichuan Vivant Biotech Co., Ltd., China |
| Nucleic acid extraction kit | QIAamp DNA Blood Mini Kit, Qiagen |
| PCR primers (T. Whpplei) | Shanghai Bioscienceres Co., Ltd., China |
| PCR amplification | TB Green Premix Ex Taq, Takara, Dalian, Liaoning, China |

| **Devices** | **Manufacturers/Models** |
| --- | --- |
| Ultrasound system | Mindray Medical International Co., Ltd., Shenzhen, China/Resona R9s |
| 18F-fluorodeoxyglucose positron emission tomography (18F-FDG PET) scanner | Philips, the Netherlands/Ingenuity TF |
| Computed tomography (CT) scanner | General Electric (GE), USA/Lightspeed VCT |
| Thoracoscope | Olympus, Japan/LTF-240 |
| laparoscope | Olympus, Japan/LTF-240 |
| Cardiac ultrasound system | Philips, the Netherlands/epic7c |
| Magnetic resonance imaging (MRI) scanner | General Electric (GE), USA/Discovery MR750W |
| Light microscope | Olympus, Japan/BX43 |
| Polymerase chain reaction (PCR) amplifier | Roche Diagnostics (Shanghai) Co., Ltd., China |
| Sanger sequencer | Thermo Fisher Scientific Applied Biosystems/QuantStudio5 System |
